# Missed opportunities for earlier diagnosis of HIV infection in people living with HIV in Thailand

**DOI:** 10.1371/journal.pgph.0000842

**Published:** 2022-07-29

**Authors:** Angsana Phuphuakrat, Kanitin Khamnurak, Sirawat Srichatrapimuk, Wittaya Wangsomboonsiri

**Affiliations:** 1 Department of Medicine, Faculty of Medicine Ramathibodi Hospital, Mahidol University, Bangkok, Thailand; 2 Chakri Naruebodindra Medical Institute, Faculty of Medicine Ramathibodi Hospital, Mahidol Univerisity, Samut Prakan, Thailand; 3 Department of Medicine, Sawanpracharak Hospital, Nakhon Sawan, Thailand; University of California Irvine, UNITED STATES

## Abstract

HIV testing is the first step to making people living with HIV (PLHIV) aware of their status. Thailand is among the countries where antiretroviral therapy is initiated in PLHIV at the lowest CD4 cell counts. We aimed to quantify and characterize missed opportunity (MO) for earlier diagnosis of HIV infection in PLHIV in Thailand. The medical records of adults who were newly diagnosed with HIV between 2019 and 2020 at the two tertiary hospitals in Thailand were reviewed. A hospital visit due to an HIV clinical indicator disease but an HIV test was not performed was considered an MO for HIV testing. Of 422 newly diagnosed PLHIV, 60 persons (14.2%) presented with at least one MO, and 20 persons (33.3%) had more than one MO. In PLHIV with MO, the median (interquartile range) time between the first MO event and HIV diagnosis was 33.5 (7–166) days. The three most common clinical manifestations that were missed were skin manifestations (25.0%), unexplained weight loss (15.7%), and unexplained lymphadenopathy (14.3%). Anemia was a factor associated with MO for HIV diagnosis [odds ratio (OR) 2.24, 95% confidence interval (CI) 1.25–4.35; *p* = 0.018]. HIV screening reduced the risk of MO for HIV diagnosis (OR 0.53 95% CI 0.29–0.95; *p* = 0.032). In conclusion, MOs for earlier diagnosis of HIV infection occurred in both participating hospitals in Thailand. Skin manifestations were the most common clinical indicator diseases that were missed. HIV testing should be offered for patients with unexplained anemia. Campaigns for HIV screening tests should be promoted.

## Introduction

Treatment of human immunodeficiency virus (HIV) infection by antiretroviral therapy (ART) has reduced morbidity and mortality in people living with HIV (PLHIV) and prevented the risk of onward transmission. HIV testing and diagnosis is the first step to making PLHIV aware of their status and entering the cascade of care. Despite the free HIV testing programs supported by the government and non-governmental organizations in Thailand, it was estimated that 50% of Thai PLHIV did not aware of their HIV status and 50% of Thai PLHIV started ART at CD4 counts below 100 cells/mm^3^ [1]. Late detection of HIV infection leads to delayed treatment and results in increased morbidity and mortality from acquired immunodeficiency syndrome (AIDS), healthcare costs [2], and treatment complexity.

Thailand has been scaling up ART. The national guidelines on HIV/AIDS treatment and prevention have recommended treating all PLHIV since 2014, irrespective of CD4 cell count. Access to HIV care and treatment is also free of charge in government hospitals. However, the proportion of PLHIV with late ART initiation was still significant. A previous study reported a global trend of increased median CD4 cell counts at the start of ART in PLHIV who started ART between 2002 and 2015 [3]. Notwithstanding this, Thailand was among the countries in which ART was commenced at the lowest CD4 spectrum [4], and approximately 75% of PLHIV started ART with CD4 cell count <200 cells/mm^3^ [3]. At Ramathibodi Hospital, the medians CD4 cell count at HIV diagnosis during 2011–2013, 2015–2017, and 2018 were 146 (45–298) [5], 159 (53–308) [6], and 116 (34–332) [7] cells/mm^3^, respectively.

There has been no study on the risk factors of late HIV diagnosis in Thailand. In this study, we quantified and characterized missed opportunity (MO) for earlier diagnosis of HIV infection in PLHIV in Thailand. We also studied the risk factors of the MO for earlier diagnosis of HIV infection.

## Materials and methods

### Study setting

This study was performed at Ramathibodi and Sawanpracharak hospitals in Thailand. Ramathibodi Hospital is a 1300-bed university hospital in Bangkok that serves a diverse population in central Thailand and is capable of super tertiary care. Sawanpracharak Hospital is a 700-bed tertiary hospital, which serves as a primary-level community hospital for Nakhon Sawan province and as a secondary and tertiary referral hospital for upper central Thailand. We retrospectively reviewed medical records of persons who were newly diagnosed with HIV infection between January 2019 and December 2020 by retrieving from the hospital databases. We included PLHIV whose age was at least 15 years old. PLHIV with previously known HIV-positive before 2019 were excluded. Sociodemographic data (e.g., gender, age at HIV diagnosis, HIV transmission route, and race) and clinical as well as laboratory data (e.g., CD4 cell counts, HIV viral load, clinical manifestations, and AIDS-defining illness at diagnosis) were included in the analyses.

The study protocol was reviewed and approved by the Ethical Clearance Committee on Human Rights Related to Research Involving Human Subjects of the Faculty of Medicine Ramathibodi Hospital, Mahidol University (MURA2021/64) and the Research and Journal Division of Sawanpracharak Hospital (23/2564). As the study was retrospective, the ethics committees waived the requirement for informed consent.

### Definitions

An MO was defined as a healthcare encounter due to a clinical manifestation that may be caused by HIV infection or as a healthcare encounter due to an AIDS-defining illness (clinical indicator disease) but HIV was not tested [8]. AIDS-defining illnesses were defined following Thailand National Guidelines on HIV/AIDS treatment and prevention 2017 [1]. Clinical indicator diseases for adult HIV infection were recorded following the UK National Guidelines for HIV testing 2008 [9] and Australian National HIV Testing Policy 2011 [10]. Late presenters were defined as PLHIV whose CD4 cell count at HIV diagnosis was less than 350 cells/mm^3^ [11]. HIV screening included an HIV test performed in a check-up program, preoperative screening, during antenatal care, before entry to inpatient care, before entry to prison, following the positive result of the partner, and according to the patient’s request. Anemia was defined following World Health Organization (WHO) criteria for anemia (hemoglobin <13 g/dL in men and <12 g/dL in women) [12].

### Statistical methods

All data were retrieved from patients’ medical records and were compiled in a tabular manner, using an Excel datasheet (see S1 Data). Statistical analyses were performed using Stata statistical software version 17.0 (StataCorp, College Station, TX, USA). Mean±standard deviation or median (interquartile range; IQR) were used to describe continuous variables. Student’s *t*-test, Mann-Whitney *U*-test, chi-square test, and Fisher’s exact test were used for the comparisons, as appropriate, with the level of significance set at a *p*-value of <0.05. A logistic regression model was performed to determine factors associated with MO of earlier diagnosis of HIV infection. Variables that presented a *p*-value of <0.2 from univariable logistic regression, or those that may affect missing opportunities despite their *p*-values of more than 0.2 were considered in a multivariable logistic regression model. Odds ratio (OR) and its 95% confidence interval (CI) were estimated.

## Results

### Patient characteristics

During the study period, we identified 422 persons newly diagnosed with HIV infection (231 and 191 persons at Ramathibodi and Sawanpracharak hospitals, respectively). The mean age at the diagnosis was 39.3±13.4 years, and 290 persons (68.7%) were men. Nearly all (98.6%) were Thai. Of these, 340 persons (80.6%) had available CD4 cell count data. The median CD4 at HIV diagnosis was 160 (44–313) cells/mm^3^. At the diagnosis, 275 persons (80.9%) were late presenters and 196 persons (57.7%) had CD4 cell counts less than 200 cells/mm^3^. The most common HIV transmission routes were heterosexual (61.1%) followed by homosexual (23.3%). The most common reason for testing was physician request (181 persons; 42.9%) and patients were mostly diagnosed in an outpatient care setting (253 persons; 60.0%). Nearly half of the PLHIV (205 persons, 48.6%) had clinical indicator diseases at an HIV diagnosis.

Sixty patients (14.2%) had presented at least one MO. Table 1 shows characteristics of PLHIV at HIV diagnosis stratified by history of MO. There were no significant differences in age, gender, or body mass index (BMI) among PLHIV with or without MOs. There was a higher proportion of PLHIV with MO in persons whose risk was heterosexual, but the difference was not statistical significance (*p* = 0.424).

**Table 1:** Characteristics of newly diagnosed PLHIV at HIV diagnosis categorized by a history of missed opportunities for earlier HIV diagnosis.

	PLHIV without MO (n = 362)	PLHIV with ≥1 MO (n = 60)	*p*-value
Age, (years), mean±SD	38.9±13.1	41.5±15.2	0.118
Male, n (%)	253 (69.9)	37 (61.7)	0.203
Body mass index, (kg/m^2^) mean±SD	21.9±4.3	21.2±3.4	0.253
Hospital, n (%)			0.426
Ramathibodi	201 (55.5)	30 (50.0)	
Sawanpracharak	161 (44.5)	30 (50.0)	
Race, n (%)			0.315
Thai	356 (98.3)	60 (100.0)	
Foreigners	6 (1.7)	0 (0.0)	
HIV risk			0.424
Heterosexual	215 (59.4)	43 (71.7)	
Homosexual	98 (27.1)	13 (21.7)	
Bisexual	1 (0.3)	0 (0.0)	
Persons who inject drugs	1 (0.3)	0 (0.0)	
Unknown	47 (13.0)	4 (6.7)	
Clinical indicator diseases			0.003
No	198 (54.7)	19 (31.7)	
Disease may be caused by HIV infection	97 (26.8)	27 (45.0)	
AIDS-defining illness	67 (18.5)	14 (23.3)	
Reason for testing			0.065
Preoperative laboratory test	63 (17.4)	9 (15.0)	
Check-up program	4 (1.1)	0 (0.0)	
Antenatal care	13 (3.6)	0 (0.0)	
Screening on entry to inpatient care	77 (21.3)	14 (23.3)	
Screening on entry to prison	2 (0.6)	1 (1.7)	
Partner positive result	19 (5.3)	0 (0.0)	
Patient request	37 (10.2)	2 (3.3)	
Physician request	147 (40.6)	34 (56.7)	
Prior HIV test	18 (5.0)	1 (1.7)	0.496
Site of testing			0.308
Emergency department	50 (13.8)	5 (8.3)	
Inpatient care	100 (27.6)	14 (23.3)	
Outpatient care	212 (58.6)	41 (68.3)	
Ordering physician at HIV diagnosis			0.153
General practitioner	72 (19.9)	7 (11.7)	
Resident	108 (29.8)	14 (23.3)	
Fellow	13 (3.6)	4 (6.7)	
Specialist	169 (46.7)	35 (58.3)	
Hemoglobin, (g/dL), mean±SD	11.9±2.5	11.1±2.3	0.011
Anemia	197 (54.4)	46 (76.7)	0.001
WBC, (cells/mm^3^), median (IQR)	6,800 (5,310–8,520)	5,800 (4,750–7,410)	0.013
CD4, (cells/mm^3^), median (IQR)	187 (45–332)	84 (40–161)	0.003
CD4 cell counts <350 cells/mm^3^	225 (62.2)	50 (83.3)	<0.001
Platelets, (/mm^3^), mean±SD	261,939±113,501	262,644±87,842	0.964
Aspartate aminotransferase	33 (23–54)	37 (23–53)	0.781
Alanine aminotransferase	25 (15–41)	25 (16–37)	0.445
Estimated glomerular filtration rate	101.5±26.5	102.5±22.8	0.807

Abbreviations: AIDS, acquired immunodeficiency syndrome; HIV, human immunodeficiency virus; IQR, interquartile range; WBC, white blood cells.

The presence of clinical indicator diseases was different in PLHIV who had or did not have MO (Table 1). A higher proportion of PLHIV with at least one MO had clinical indicator diseases at HIV diagnosis (*p* = 0.003). In PLHIV with clinical indicator diseases, HIV testing was requested by a physician in 167 persons (81.5%). In total, HIV testing was requested by physicians in 181 persons (42.9%). Other reasons for testing were due to screening purposes. Of note, Sawanpracharak Hospital, but not Ramathibodi Hospital, has a mandatory HIV screening test on entry to inpatient care. This was able to detect 91 (21.6%) new cases during the study period. In PLHIV without clinical indicator diseases, HIV screening tests detected a significant proportion of this population, compared to those detected by physician request (93.6% vs. 0.5%; *p* <0.001).

Only 19 PLHIV (4.5%) in this study had prior HIV testing, and there was no significant difference in the proportion between the two groups. Sites of testing and physicians who requested anti-HIV tests at HIV diagnosis were not significantly different between the two groups.

The proportion of late presenters was significantly higher in PLHIV with a history of MO (83.3% vs. 62.2%; *p* <0.001). In PLHIV with and without MO, the median CD4 cell counts were 84 (40–161), and 187 (45–332) cells/mm^3^, respectively. CD4 cell counts were significantly higher in PLHIV without history of MO (*p* = 0.003). White blood cells were also significantly higher in PLHIV without history of MO (6,800 vs. 5,800 cells/mm^3^; *p* = 0.013). Hemoglobin levels were higher in PLHIV without history of MO (11.9 vs. 11.1; *p* = 0.011). There were no statistically significant differences in the numbers of platelets, levels of aspartate aminotransferase, alanine aminotransferase, and estimated glomerular filtration rate (Table 1).

### Missed opportunity events and clinical indicator diseases

**Fig 1:**
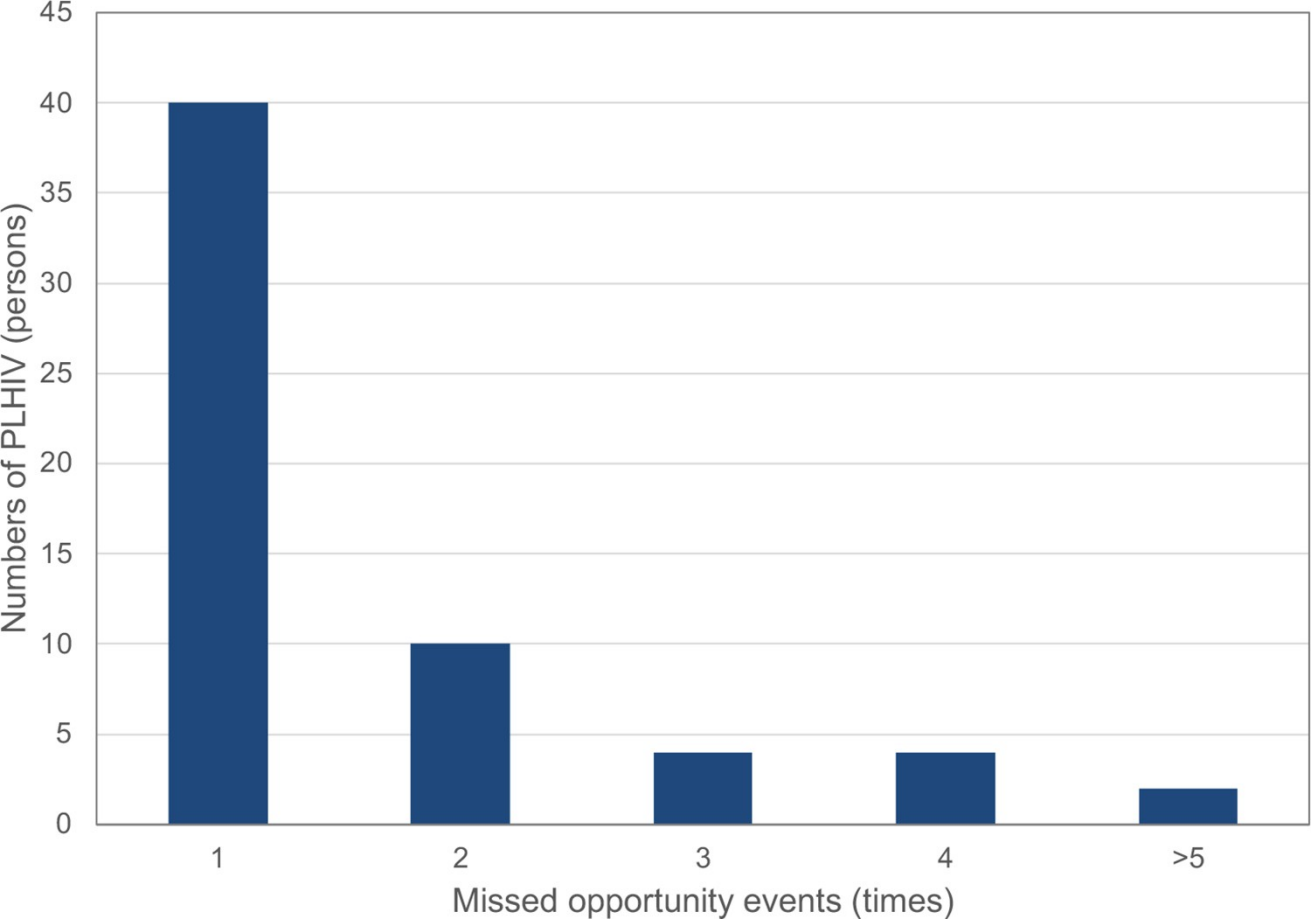
Numbers of missed opportunity events for earlier diagnosis of HIV infection in PLHIV at Ramathibodi and Sawanpracharak hospitals between 2019 and 2020.

Of the 60 PLHIV who had at least one MO, 20 persons (33.3%) had more than one MO (Fig 1). The median duration between the first MO event and HIV diagnosis was 33.5 (IQR 7–166, range 1–1,472) days. The median number of MO event was one event per person (IQR 1–2, range 1–22).

There were 117 MO events and 140 clinical indicator diseases in PLHIV with a history of MO. Of the clinical indicator diseases, four diseases (2.9%) were AIDS-defining illnesses. Table 2 shows clinical indicator diseases that were missed in the study. The most common clinical indicator diseases that were missed included skin manifestations (25.0%), unexplained weight loss (15.7%), and unexplained lymphadenopathy (14.3%) respectively (Table 2). For the skin manifestations, dermatitis/seborrheic dermatitis was the most common skin manifestation that HIV testing should be offered but missed. In PLHIV who had unexplained weight loss, pulmonary tuberculosis was the most common final diagnosis. In those who had unexplained lymphadenopathy, pathological diagnosis revealed reactive lymphadenopathy and lymphoma as the most common final diagnosis.

**Table 2:** Numbers of clinical indicator diseases that were missed in the study.

Clinical manifestations	Number (%)
Skin manifestations	35 (25.0)
Dermatitis / seborrheic dermatitis	28 (20.0)
Folliculitis	2 (1.4)
Eczema	5 (3.6)
Unexplained weight loss	22 (15.7)
Unexplained lymphadenopathy	20 (14.3)
Cervical dysplasia	11 (7.9)
Herpes zoster in an individual <50 years old	9 (6.4)
Sexually transmitted infection	7 (5.0)
Unexplained leukocytopenia/thrombocytopenia persisting for >4 weeks	7 (5.0)
Chronic cough	6 (4.3)
Anal wart	5 (3.6)
Unexplained oral candidiasis	3 (2.1)
Subacute fever	3 (2.1)
Prolonged fever	3 (2.1)
Pulmonary tuberculosis	2 (1.4)
Disseminated or extrapulmonary tuberculosis	1 (0.7)
Hepatitis B	1 (0.7)
Lymphoma	1 (0.7)
Peripheral neuropathy of unknown origin	1 (0.7)
Unexplained chronic diarrhea	1 (0.7)
Tonsillitis (recurrent)	1 (0.7)
Infectious mononucleosis	1 (0.7)

### Risk factors of missed opportunities for earlier HIV diagnosis

Univariable and multivariable analyses of factors associated with an MO for earlier diagnosis of HIV infection were performed (Table 3). In univariable analysis, HIV screening and anemia were associated with MO. In multivariable analysis, anemia showed an increased risk of MOs (OR 2.24; 95% CI 1.15–4.35; *p* = 0.018). HIV screening reduced the risk of MO for HIV diagnosis (OR 0.53; 95% CI 0.29–0.95; *p* = 0.032).

**Table 3:** Univariable and multivariable analyses of factors associated with missed opportunity for earlier diagnosis of HIV infection.

Factors	Univariable analysis			Multivariable analysis		
	OR	95% CI	*p-*value	OR	95% CI	*p-*value
Age	1.02	1.00–1.04	0.119	1.01	0.98–1.03	0.582
Female	1.44	0.82–2.54	0.205	1.07	0.54–2.12	0.843
Heterosexual	1.73	0.95–3.15	0.073	1.64	0.77–3.48	0.197
HIV screening test	0.52	0.30–0.91	0.021	0.53	0.29–0.95	0.032
Specialist request	1.60	0.92–2.78	0.096	1.70	0.96–3.01	0.068
Anemia	2.75	1.46–5.18	0.002	2.24	1.15–4.35	0.018
Leukopenia	2.03	0.91–4.53	0.085	1.61	0.70–3.71	0.263

Abbreviations: CI, confidence interval; OR, odds ratio.

## Discussion

This study demonstrated 14.2% of MOs for earlier diagnosis of HIV infection occurred in our hospitals. In PLHIV who had at least one MO had lower CD4 count, and higher proportions of PLHIV with clinical indicator diseases at HIV diagnosis compared to those who did not have an MO. In this study, skin manifestations were the most common clinical indicator diseases that were missed. Anemia was significantly associated with an increased risk of MO, whereas HIV screening reduced the risk of MO.

A previous study showed that median CD4 cell counts at ART initiation increased over time in Asian PLHIV from 2007 to 2011 [13]. In our study, however, we revealed approximately 80% of newly diagnosed PLHIV in Thailand were presented late. The proportion of late presenters in Thailand was higher than in other countries in Asia and the Pacific [14]. Previous studies showed 43% of PLHIV in China [15] and 63% of those in Malaysia [16] had a late presentation. Older age, male gender, and persons who inject drugs were factors associated with a presentation into care at CD4 cell count <200 cells/mm^3^ in Asian countries [17].

Factors associated with MO were different in various settings. In Israel, a low prevalence setting, there were no national guidelines concerning HIV testing except for the guidelines regarding pregnant women. Approximately one-third of PLHIV in the Israel study were diagnosed late; old age and heterosexual were the risk factors. Hematological diseases were the most common clinical indicator diseases that were missed in the study [18]. A study from Europe in the setting of an HIV outpatient clinic in Switzerland showed that 59% of newly diagnosed PLHIV were late presenters, and 47% had presented with at least one MO [11]. In this setting, MO was associated with individuals from sub-Saharan Africa, men who have sex with men, and patients under follow-up for chronic disease. In the United Kingdom, the study of MO in newly diagnosed HIV infection in Africans revealed that nearly half of the participants were diagnosed with HIV infection when CD4 cell count was <200 cells/mm^3^ [19]. Approximately 75% of the participants attended general practitioner in the two years before HIV diagnosis. However, HIV testing was not offered for 82.4% of those who accessed the services in the year before HIV diagnosis. A study in Malaysia reported 57% of MOs for earlier HIV diagnosis [16]. Unexplained fever and/or fever lasting more than one month were the most common presenting symptom that was missed. Our study showed skin manifestations were the most common clinical indicator disease that were missed.

Multivariable analysis in our study demonstrated unexplained and/or unaware anemia was associated with MOs for earlier HIV diagnosis. Anemia was identified as a factor related to MOs for earlier HIV diagnosis in Canada [20]. In the study, 21%, 19%, and 18% of anemia was related to iron deficiency anemia, vitamin B12 deficiencies, and unspecified causes, respectively. We identified an HIV screening test as a factor that reduced the risk of MOs, this factor agreed with the earlier study [21]. Of note, HIV screening on entry to inpatient care could detect many new cases in this study.

Thailand National Guidelines recommend HIV testing in persons who had signs and symptoms compatible with HIV infection or AIDS [1], but the clinical indicators of HIV infection were not described in detail. Adding the description of clinical indicator diseases to the guidelines might help remind clinicians when HIV testing should be offered. Unexplained anemia should be added to the conditions that anti-HIV should be tested. Thailand National Guidelines recommend HIV testing following the WHO guidelines on HIV testing services [22], in which counseling and consent are needed before HIV testing. In 2006, the United States Centers for Disease Control and Prevention (CDC) revised the HIV testing guidelines. The current CDC guidelines recommend an opt-out HIV screening in health-care settings [23]. This strategy does not require neither specific signed consent for HIV testing nor prevention counseling. HIV screening is included in the standard screening tests in all health-care settings unless the patient declines. The randomized clinical trial performed at the emergency department of a teaching hospital and regional trauma center showed that this opt-out approach significantly increased HIV testing acceptance [24]. In Thailand, the opt-out screening was previously studied in the setting of women undergoing treatment for cervical neoplasia with 100% patient acceptance [25]. This suggested the feasibility of the opt-out approach implementation in health-care settings, e.g., screening before entry to inpatient care, in Thailand.

The strength of this study is that we compiled the newly diagnosed PLHIV in two hospitals located in different regions and had different screening programs. This study had some limitations. We were able to review the medical records only in these hospitals, and the patients might visit outside hospitals or services before presenting to the participating hospitals. Moreover, some data were incomplete, as with any retrospective study.

In conclusion, MOs for earlier diagnosis of HIV infection occur in both hospitals in Thailand and a large proportion of PLHIV presented late. Skin manifestations were the most common clinical indicator diseases that were missed. Unexplained and/or unaware anemia was associated with MOs for earlier HIV diagnosis, whereas HIV screening tests decreased the risk. HIV screening programs should be promoted, and the national guidelines should provide the details on unexplained anemia and clinical indicator diseases that HIV testing should be offered.

## Supporting information

S1 Data Excel file with raw data that was used for all analysis shown in the paper. (XLSX)
